# CD101: a novel long‐acting echinocandin

**DOI:** 10.1111/cmi.12640

**Published:** 2016-07-22

**Authors:** Yanan Zhao, Winder B. Perez, Cristina Jiménez‐Ortigosa, Grayson Hough, Jeffrey B. Locke, Voon Ong, Ken Bartizal, David S. Perlin

**Affiliations:** ^1^ Public Health Research Institute, Rutgers Biomedical and Health SciencesNew Jersey Medical SchoolNewarkNJUSA; ^2^ Cidara Therapeutics, Inc.San DiegoCAUSA

## Abstract

CD101 is a novel echinocandin drug being developed to treat severe fungal infections including invasive candidiasis. We have performed a series of studies to evaluate the antifungal properties of CD101 against both echinocandin‐susceptible and ‐resistant *Candida* strains. Antifungal susceptibility testing performed on a collection of 95 *Candida* strains including 30 caspofungin‐resistant isolates containing *fks* mutations demonstrated comparable antifungal potency of CD101 relative to micafungin (MCF) across different *Candida* species. Comparable kinetic inhibition of glucan synthase activity was also observed for CD101 and MCF on both wild‐type (WT) and resistant *fks* mutant *Candida* strains. Similarly, both drugs yielded nearly identical values for a mutant prevention concentration. In a murine model of invasive candidiasis, CD101 displayed better or at least comparable efficacy relative to MCF in treating WT or *fks* mutant *Candida albicans*. An exceptional long‐lived pharmacokinetic profile was observed in mice following a single dose of CD101. Collectively, CD101 has great potential not only in treating invasive *Candida* infections but also in preventing emergence of resistance to currently approved echinocandin drugs.

## Introduction

The echinocandin drugs are the first class of antifungals to target the fungal cell wall (Hector, 1993). These compounds are potent inhibitors of the catalytic subunit of β‐1,3‐D‐glucan synthase, which is responsible for biosynthesis of β‐1,3‐D‐glucan, the major fungal cell wall biopolymer (Denning, 2003). The echinocandins demonstrate *in vitro* fungicidal activity against most *Candida* species, including azole‐resistant yeasts, and they are recommended as first‐line therapy for both nonneutropenic and neutropenic patients with candidemia. Echinocandins also are the preferred empiric therapy for suspected candidiasis in nonneutropenic patients in the intensive care unit (Pappas *et al.,* 2009; Pappas *et al.,* 2016). Overall, current frequency of echinocandin resistance remains relatively low (< 1%) with *Candida albicans* and most other *Candida* species except *Candida glabrata* (Castanheira *et al.,* 2010; Pfaller *et al.,* 2011; Pfaller *et al.,* 2013). However, widespread echinocandin usage has been accompanied by reports of emerging multidrug resistance among clinical *Candida* isolates (Alexander *et al.,* 2013; Fekkar *et al.,* 2014), as well as epidemiological shifts with increased proportion of less susceptible *Candida* species (Lortholary *et al.,* 2011). Resistance to echinocandins is associated with mutations in two hot spot (HS) regions in the *FKS* genes that correlate with clinical failure or poor response to therapy (Perlin, 2011; Shields *et al.,* 2012; Beyda *et al.,* 2014).

**Figure 1:**
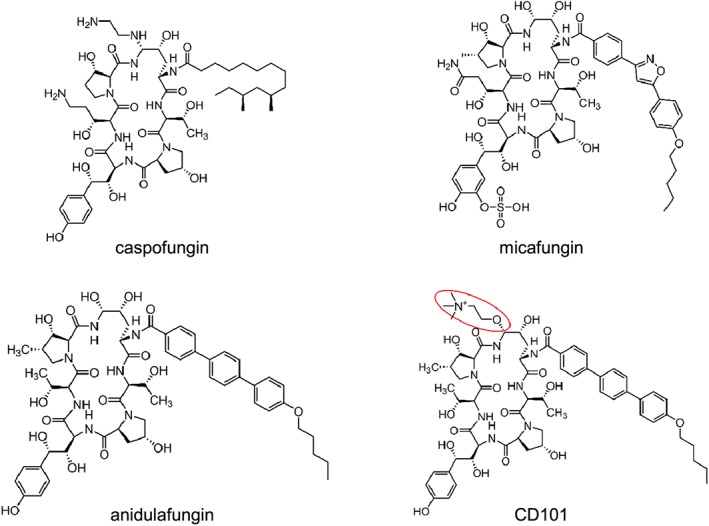
Chemical structure of echinocandin drugs.

As a novel echinocandin drug candidate, CD101 has a modified structure (Fig. 1) that confers both superior pharmacokinetics (PK) properties and the potential for an improved safety profile relative to other drugs in the same class (Ong *et al.,* 2015b; Ong *et al.,* 2015a; Rubino *et al.,* 2015). CD101 has demonstrated potent *in vitro* activity against a broad range of *Candida* and *Aspergillus* species including some antifungal‐resistant strains (Castanheira *et al.,* 2014). Presently, a paucity of information exists concerning the antifungal properties of CD101 against well‐defined echinocandin‐resistant clinical isolates. Hence, we have performed a series of studies to comprehensively evaluate antifungal properties of CD101 against both echinocandin‐susceptible and ‐resistant *Candida* strains, from *in vitro* susceptibility, enzyme activity assessment, mutant prevention assay, to *in vivo* PK study and efficacy evaluation against echinocandin‐susceptible and ‐resistant *C*. *albicans* strains in a mouse model of invasive candidiasis.

## Results

### Minimal inhibitory concentration (MIC) distributions

Antifungal susceptibility testing was performed for a collection of 95 *Candida* strains (20 *C*. *albicans*, 20 *C*. *glabrata*, 2 *Candida dubliniensis*, 15 *Candida krusei*, 19 *Candida parapsilosis* and 19 *Candida tropicalis*) that included 30 *fks* mutant isolates showing a caspofungin‐resistant phenotype as per Clinical and Laboratory Standards Institute (CLSI) interpretive criteria (CLSI, 2012). The MIC distributions of the *Candida* isolates for micafungin (MCF) and CD101 are shown in Table 1. CD101 did not show appreciable differences in MICs for the wild‐type (WT) isolates relative to MCF with the exception of *C*. *krusei* isolates, for which MICs were two‐ to fourfold lower for CD101 than MCF. As for caspofungin resistant isolates, CD101 MICs were also generally comparable with that of MCF.

**Table 1:** MIC distributions of MCF and CD101 for the *Candida* isolates included in this study.

	Phenotype^a^ (no. of isolates)	Micafungin	CD101
Species		MIC_50_ mode value [range (µg/ml)]	MIC_50_ mode value [range (µg/ml)]
*C*. *albicans*	WT (10)	≤0.03 (≤0.03)	≤0.03 (≤0.03)
	CR (10)	1 (0.03–4)	2 (0.12–2)
*C*. *glabrata*	WT (9)	≤0.03 (≤0.03)	0.06 (≤0.03–0.06)
	CR (11)	0.06/0.25 – 2/4 (0.06–4)	1 (0.12–4)
*C*. *dubliniensis*	WT (1)	0.03	0.03
	CR (1)	0.03	0.03
*C*. *krusei*	WT (11)	0.12 (0.03–0.25)	≤0.03 (≤0.03–0.06)
	CR (4)	0.03 (0.03–8)	≤0.03–4 (≤0.03–4)
*C*. *parapsilosis*	WT (19)	4 (2–8)	2 (2–4)
*C*. *tropicalis*	WT (15)	0.03 (0.03)	0.03 (0.03)
	CR (4)	2 (1–2)	2 (0.25–2)

a CR, caspofungin resistant.

### IC_50_s for C. albicans and C. glabrata isolates

To better assess direct inhibition of CD101 on glucan synthase, the kinetic inhibition parameter IC_50_ (half‐maximal inhibitory concentration) was determined for glucan synthases from WT and *fks* mutant *Candida* strains. The inhibition curves for MCF and CD101 against the *C*. *albicans* WT isolate showed the typical pattern of β‐1,3‐D‐glucan synthase echinocandin susceptibility reported with other sensitive *Candida* species (Park *et al.,* 2005; Garcia‐Effron *et al.,* 2009), with mean IC_50_s of 17.7 and 14.3 ng/ml for MCF and CD101, respectively (supplementary figure and Table 2). Decreased echinocandin susceptibility was observed with the resistant *C*. *albicans* F641S, S645P and S645P/S mutant enzymes. The F641S mutant (DPL18) exhibited a 100‐fold and 24‐fold increase in IC_50_s for MCF and CD101, respectively, compared to the WT. The S645P (DPL20) and S645P/S mutant (DPL22) exhibited 144‐, 14‐fold and 185‐, twofold increase for MCF and CD101 respectively. Whereas the IC_50_s for MCF and CD101 were similar for the S645P mutant, the IC_50_ for CD101 was approximately fivefold lower than the MCF IC_50_ for the F641S mutant. Mean IC_50_ values for the WT *C*. *glabrata* enzyme were 0.5 and 2.6 ng/ml for MCF and CD101, respectively (supplementary figure). The *C*. *glabrata* F659del mutant glucan synthase did not exhibit appreciable reductions in activity after treatment with a high concentration (10 000 ng/ml) of either MCF or CD101. However, the S663P mutant exhibited a lower IC_50_ for MCF compared to CD101.

**Table 2:** Half maximal inhibitory concentration (IC_50_) values for susceptible and resistant *C*. *albicans* and *C*. *glabrata* isolates used in the study.

		Fks mutation		IC_50_ (ng/ml)^a^	
Strain	Species	Fks1p	Fks2p	MCF	CD101
DPL1002	*C*. *albicans*	WT	—	17.7	14.3
DPL18	*C*. *albicans*	F641S	—	1782.0	347.4
DPL20	*C*. *albicans*	S645P	—	2555.8	2641.4
DPL22	*C*. *albicans*	S645P/S	—	245.5	30.5
DPL50	*C*. *glabrata*	WT	WT	0.5	2.6
DPL23	*C*. *glabrata*	WT	F659del	> 10 000	> 10 000
DPL30	*C*. *glabrata*	WT	S663P	6772.3	> 10 000

a IC_50_s are the arithmetic mean of three replicate determinations.

### Mutant prevention concentration (MPC) determination

**Figure 2:**
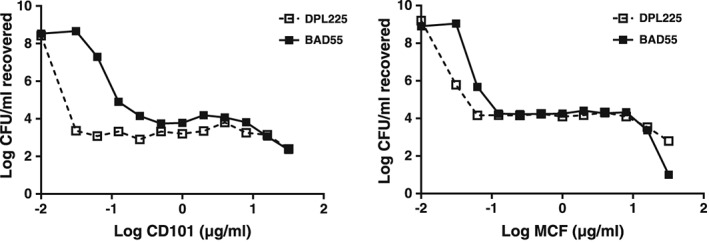
CD101 and MCF MPC determination for wild‐type *C*. *albicans* and *C*. *glabrata strains*. Fungal cell suspension containing 1 × 10^7^ CFU of WT *C*. *albicans* (DPL225) or WT *C*. *glabrata* (BAD55) were treated with CD101 or MCF ranging from 0.03 to 32 µg/ml for 24 h. Colonies recovered from treatment were measured by quantitative culture. MPC was defined as the concentration where the second sharp decline of colony counts occurred on the concentration–CFU curve.

The concept of MPC was developed to determine the concentration of antibiotic to prevent the development of resistant bacterial isolates. The MPC is the minimal concentration that suppresses the adapted subpopulation which persists above MIC drug levels, and it is a measure of the susceptibility of the adapted and potentially resistant mutant population (Dong *et al.,* 1999; Zhao and Drlica, 2001). Therefore, administering antibiotic at a dose above the MPC would inhibit the growth of potentially resistant isolates. Using a modified version of the original method, we observed a 3‐ to 5‐log decrease in CFUs around the MICs for MCF and CD101 and a second sharp decrease of recovered colony counts at around 16–32 µg/ml (Fig. 2). We determined the MPC for both MCF and CD101 to be 16 µg/ml against testing WT *C*. *albicans* (DPL225) and *C*. *glabrata* (BAD55) strains.

### In vivo efficacy of CD101 to treat echinocandin resistant C. albicans in a mouse model of invasive candidiasis

**Figure 3:**
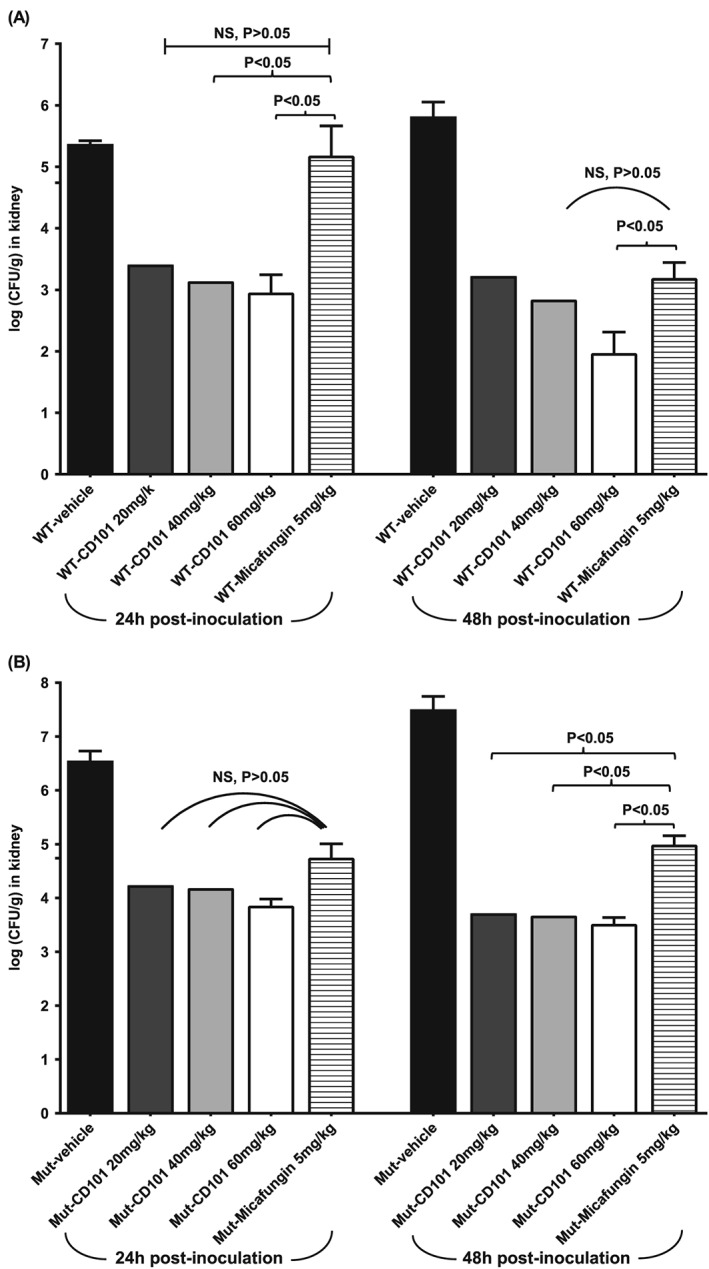
Kidney burden comparison among different treatment groups of mice infected with (A) *C*. *albicans* WT strain ATCC 90028 or (B) *C*. *albicans* mutant strain DPL22 S645P/S at 24 h and 48 h post‐infection. Each bar represents the mean burden in the kidneys from five mice. The error bars represent standard deviations.

**Figure 4:**
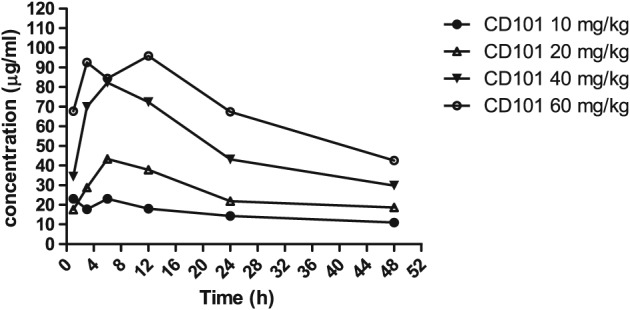
Mean plasma concentration–time profiles of CD101 following single intraperitoneal administration of CD101 at 10, 20, 40 and 60 mg/kg in infected immunocompetent mice. Each symbol represents the mean plasma drug concentration determined for three mice.

To evaluate the *in vivo* efficacy of CD101 against echinocandin resistant *Candida* strain, we compared kidney burdens following single dosage of CD101 treatment in neutropenic mice systemically infected with either a WT or resistant *C*. *albicans* mutant strain with a heterozygous *fks*/*FKS* S645P/S modification (MIC ranged from 0.12 to 0.5 in triplicate testing for both MCF and CD101). CD101 at three increasing dosages of 20, 40 and 60 mg/kg (based on mice PK and human phase 1 clinical trial data, these doses are projected to approximate a range of plasma exposures in mice that would include the actual once‐weekly clinical CD101 dose selected), and MCF at 5 mg/kg, equivalent to human therapeutic dosage, were included in this evaluation. CD101 at all three testing dosages strongly exhibited activity against both WT and *fks*/*FKS* mutant strains of *C*. *albicans*, as demonstrated by significant kidney burden reduction in all treatment groups at both 24 h and 48 h post‐inoculation time points (*P* < 0.05) (Figs 3A and 4B). In WT strain infected mice, CD101 exhibited better efficacy than MCF at 24 h post‐inoculation at all three doses (20 mg/kg dose did not achieve statistical significance by post hoc test possibly because of the relatively large variance within the group and small group sample size. Nevertheless, an almost 2 log mean burden difference between 20 mg/kg CD101 and 5 mg/kg MCF was observed). Although the observed superiority of CD101 relative to MCF was only seen with the highest dose (60 mg/kg) at 48 h post‐inoculation, the efficacy of CD101 at 20 mg/kg and 40 mg/kg was still comparable with MCF at 5 mg/kg (Fig. 3A). Regarding the echinocandin resistant *fks*/*FKS* mutant strain infected mice, CD101 treatment significantly reduced kidney burdens by over 2 logs at 24 h post‐inoculation compared to vehicle control (*P* < 0.05). The 24 h burden reduction was not significantly different among the three CD101 dosage groups or MCF treatment group. However, better efficacy of CD101 compared to MCF at 5 mg/kg was observed for all three doses at 48 h post‐inoculation. Burden reduction was comparable between the three CD101 groups (Fig. 3B).

### PK

A single‐dose PK evaluation of CD101 was undertaken in immunocompetent mice following intraperitoneal (IP) doses of 10, 20, 40 and 60 mg/kg of CD101 at 24 h post‐systemic infection with WT *C*. *albicans* strain. The time‐course plasma levels of CD101 are shown in Fig. 4. The PK of the drug were relatively linear over the dose range. Maximum plasma concentrations (C_max_) of CD101 were observed at 1 h, 6 h, 6 h and 12 h with mean C_max_ values of 23.1, 43.3, 82.3 and 95.8 µg/ml for the doses of 10, 20, 40 and 60 mg/kg respectively. The mean values for AUC_0‐t_, where *t* = 48 h post‐dose, were 736, 1250, 2380 and 3300 µg*h/ml for the doses of 10, 20, 40 and 60 mg/kg respectively. The elimination half‐life was long for each dose, ranging from 29.8 to 52.0 h.

## Discussion

Previous *in vitro* susceptibility studies have demonstrated potency of CD101 against a wide spectrum of *Candida* species and *Aspergillus* species, variable but overall comparable to anidulafungin and caspofungin (Castanheira *et al.,* 2014). In the current study, we have assessed the *in vitro* susceptibility profile for CD101 against a well‐characterized panel of caspofungin resistant clinical isolates with *fks* mutations from a range of *Candida* species. As expected, CD101 displayed similar activity to MCF against different resistant isolates included in the testing panel, although some slight differences (twofold) between CD101 and MCF were observed in the MIC distribution pattern against *C*. *albicans* and *C*. *glabrata* ER isolates. In addition, a two to fourfold enhanced activity of CD101 relative to MCF was observed for *C*. *krusei* isolates, regardless of *FKS* genotype. These findings were consistent with previous observations (Hall *et al.,* 2015) and further support the potential of clinical use of CD101.

MIC and *FKS* genotype status are the key parameters that determine therapeutic response (Perlin *et al.,* 2015). To better understand potential drug–target interactions, we assessed the inhibitory activity of CD101 on its target glucan synthase from 3 *C*. *albicans* and 3 *C*. *glabrata* isolates with various *FKS* genotypes. Not surprisingly, the enzyme kinetic profiles were not remarkably different between CD101 and MCF, except that CD101 tends to be slightly more effective than MCF on *C*. *albicans fks* mutant enzymes whereas it is not as potent as MCF when tested against *C*. *glabrata fks* mutants. These data are consistent with the susceptibility testing results. Whether such enzyme activity variance against different *Candida* species carrying *fks* mutations has clinical implications or not will need to be further assessed.

While drug resistance has emerged with echinocandins over the last decade (Arendrup and Perlin, 2014), strategies to efficiently suppress the acquisition of resistance have not been successfully established. A key consideration to this dilemma is that it lacks proper measures of how to overcome development of resistant mutant subpopulations with antifungal drugs. The mutant selection window hypothesis was recently raised to address the critical need of dosing strategy to restrict emergence of resistance to antibacterial agents (Zhao and Drlica, 2001; Drlica and Zhao, 2007; Zhao and Drlica, 2008). This hypothesis postulates that for each antimicrobial–pathogen combination, an antimicrobial range exists in which selective amplification of single‐step, drug‐resistant mutants occurs. More specifically, the lower boundary of the mutant selection window is the lowest drug concentration that eradicates the susceptible cells, and is most likely equal to the MIC_99_, while the upper boundary of the window is the minimal concentration that inhibits drug‐susceptible mutant subpopulation, a value called the MPC. MPC is a direct measurement of the resistant mutant subpopulation susceptibility. In the present study, MPC was determined for both CD101 and MCF using WT *C*. *albicans* and WT *C*. *glabrata* strains. For both drugs, concentrations above the MIC level resulted in an adapted cell population that persisted at up to 16 µg/ml of drug level, where decrease of the persisting population was seen with further increase of drug concentration (Fig. 2). From a practical perspective, to maintain drug levels above MPC is often more stringent than necessary and increases risk of toxicity. However, the MPC‐based PK/PD measurement, AUC_24_/MPC, has been tested to be more accurate than AUC_24_/MIC in predicting resistance occurrence (Firsov *et al.,* 2006; Olofsson *et al.,* 2006; Drlica and Zhao, 2007), and *in vivo* experiments also prove the usefulness to control the frequency of resistant mutant development by maintaining drug levels above MPC for certain time of period (Croisier *et al.,* 2004; Etienne *et al.,* 2004). Given the high plasma drug exposure potential and wide safety margin of CD101, and the fact that CD101 and MCF have the same MPC value suggests a possible advantage of CD101 in preventing resistance to currently approved echinocandin drugs.

Despite a growing body of evidence demonstrating robust *in vitro* antifungal activity of CD101, little was known about the *in vivo* efficacy of CD101 against echinocandin resistant *Candida* isolates. In the current study, we partially addressed this critical question by assessing kidney burden reductions post‐antifungal treatment in mice infected with a resistant *fks*/*FKS* mutant *C*. *albicans* strain. Data acquired from this experiment suggest that CD101 has the potential to achieve efficacy against infections caused by certain *Candida* strains that are resistant to therapeutic doses of currently approved echinocandin drugs. In another comparison study in mice infected by WT *C*. *albicans* strain, the superiority of CD101 was mainly demonstrated by earlier and faster burden reduction compared to MCF (Fig. 3). The burden clearance advantage of CD101 over MCF did diminish at 48 h post‐treatment, yet it is of note that the highest testing dose of CD101 was still significantly better than MCF. The overall testing dosage of CD101 was chosen based on its safety profile and PK data in animals (Ong *et al.,* 2015b; Ong *et al.,* 2015a; Rubino *et al.,* 2015).

Echinocandins kill *Candida* cells by inhibiting β‐1,3‐D‐glucan synthase in a concentration‐dependent fashion (Douglas *et al.,* 1997; Onishi *et al.,* 2000). From a pharmacokinetic and pharmacodynamic standpoint, drugs that exhibit concentration‐dependent killing and prolonged post‐antibiotic effects are most effective when larger dose levels are administered infrequently (Vogelman *et al.,* 1988; Turnidge *et al.,* 1994; Craig, 1998). In this sense, the long elimination half‐life of CD101, as demonstrated in the current study and others (Ong *et al.,* 2015b; Rubino *et al.,* 2015), as well as its prolonged efficacy and remarkably wide safety margin (Ong *et al.,* 2015b; Ong *et al.,* 2015a), makes CD101 a promising drug candidate that achieves high plasma drug exposure with an extended interval dosing regimen, and may outcompete currently available echinocandin drugs for better prophylactic and treatment efficacy of invasive candidiasis.

In conclusion, CD101 is a novel, long‐acting echinocandin drug that exhibits both *in vitro* and *in vivo* strong antifungal activities against a wide spectrum of fungal pathogens. Given its superior PK properties, this drug has the potential to be advantageous for suppressing emergence of resistance to currently approved echinocandin drugs.

## Experimental procedures

### Strains and antifungal susceptibility testing

All strains used in this study were stocked in the Perlin lab collection. Antifungal susceptibility testing was performed in triplicate for each strain in accordance with the guidelines described in CLSI documents M27‐A3 (CLSI, 2008). *C*. *parapsilosis* ATCC 22019 and *C*. *krusei* ATCC 6258 were used as quality control strains. CD101 (Cidara Therapeutics, Inc., San Diego, CA, USA) and MCF (Astellas Pharma Inc., Tokyo, Japan) were obtained as standard powders from their manufacturer, and stock solutions were prepared by dissolving the compounds in water (MCF) or 100% dimethyl sulfoxide (DMSO; CD101).

### Glucan synthase assay

All testing strains (1 WT and 3 *fks C*. *albicans* mutant strains; 1 WT and 2 *fks C*. *glabrata* mutant strains) were grown with vigorous shaking at 37°C to early stationary phase in YPD (1% Yeast extract, 2% Peptone, 2% Dextrose) broth, and cells were collected by centrifugation. Cell disruption, membrane protein extraction and partial 1,3‐β‐D‐glucan synthase purification by product‐entrapment were performed as previously described (Garcia‐Effron *et al.,* 2009). Reactions were initiated by the addition of product‐entrapped glucan synthase. Sensitivity to MCF and CD101 was measured in a polymerization assay using a 96‐well 0.65 µm multiscreen HTS filtration system (Millipore Corporation, Bedford, MA) in a final volume of 100 µl, as previously described (Park *et al.,* 2005). Serial dilutions of the drugs (0.01–10 000 ng/ml) were used as calibration standards. MCF was dissolved in water and CD101 was dissolved in 100% DMSO. Inhibition profiles and IC_50_ values were determined using a normalized response (variable‐slope) curve fitting algorithm with GraphPad Prism, version 6.05, software (Prism Software, Irvine, CA).

### MPC determination

As originally described for bacteria, the MPC is determined by spreading a large number of cells (≥ 10^11^) on agar plates containing increasing concentrations of drug (Dong *et al.,* 1999). However, our attempts to use this method with either *C*. *albicans* or *C*. *glabrata* resulted in growth on all plates. Alternatively, *Candida* cells grown overnight in YPD broth with vigorous shaking at 37°C were collected by centrifugation and washed with distilled water. Samples were diluted to 1 × 10^8^ CFU/ml in a total volume of 1.5 ml. One hundred microlitres of fungal cell suspension was added to 0.9 ml of RPMI 1640 medium buffered with MOPS to pH 7.0 with or without drug, providing the starting inoculum of approximately 1 × 10^7^ CFU/ml. The range of CD101 or MCF concentrations tested was 0.03–32 µg/ml. The culture vials were incubated with agitation at 37°C for 24 h. A 100 µl sample was removed from each culture vial and serially diluted with sterile water. Subsequently, 100 µl aliquots of several dilutions were plated on YPD. When colony counts were suspected to be low, 100 µl was taken directly from the culture vials and plated without dilution. Plates were incubated at 37°C for 1–2 days prior to colony counting. MPC was defined as the concentration where the second sharp decline of colony counts occurred on the concentration‐CFU curve (Fig. 2).

### Animals

Female 6‐week‐old BALB/c mice (Charles River Laboratories) weighing 18–22 g were used for all animal experiments. Mice were housed in pre‐sterilized filter‐top cages and maintained in accordance with American Association for Accreditation of Laboratory Care criteria. The animal study was approved by Rutgers Institutional Animal Care and Use Committee.

### In vivo efficacy of CD101 to treat invasive candidiasis in mice

A well‐established neutropenic disseminated candidiasis murine model was used for this study (Andes, 2005). A total of 100 mice were randomized into 10 different infection/antifungal therapy arms. Sample size of this animal experiment was considered as adequate based on the ‘resource equation’ method (Charan and Kantharia, 2013). Mice were rendered neutropenic by receiving 150 mg/kg and 100 mg/kg of cyclophosphamide via IP injection on day −4 and day −1 prior to infection respectively. The organisms were subcultured in liquid YPD medium at 37°C with shaking overnight. Cells were collected by centrifugation, washed twice with sterile phosphate‐buffered saline (PBS), and counted with a haemocytometer. The inoculum was adjusted to 5 × 10^6^ CFU/ml and 100 µl was used to infect each mouse. Actual infection dose was verified by viable counts on YPD plates spread with proper dilutions of the inoculum and incubated at 37°C for 24 h. On day 0, mice were infected with 5 × 10^5^ CFU of *C*. *albicans FKS* WT (DPL1002) (*n* = 50) or heterozygous mutant strain (DPL22 S645P/S) (*n* = 50) via lateral tail vein injection. Groups of 10 mice were given single dose of vehicle (provided by Cidara Therapeutics, Inc.), CD101 at 20 mg/kg, 40 mg/kg or 60 mg/kg, or antifungal control (MCF, 5 mg/kg, equivalent to clinical therapeutic dose) at 3 h post‐infection via IP injection. At 24 h post‐inoculation and at the experiment endpoint 48 h post‐inoculation, five mice from each group were euthanized via CO_2_ inhalation and kidneys were aseptically removed for enumeration of fungal burdens. All graphic data are expressed as means ± SD and were statistically analysed by analysis of variance (ANOVA) using computer Prism software (Prism 5; GraphPad Software, Inc., San Diego, CA). Burden difference between testing and control groups was assessed by post hoc analyses, using Dunnett's or Dunn's multiple comparison test (when group values do not fit Gaussian distribution). A *P* value of < 0.05 was considered statistically significant.

### PK

The PK of CD101 was investigated using an immunocompetent mouse model of disseminated candidiasis (Wiederhold *et al.,* 2011). Mice were infected with 2.0 × 10^6^ CFU of *C*. *albicans* WT strain DPL1002 (*n* = 39) via intravenous injection. Single doses of CD101 at 10, 20, 40 or 60 mg/kg were administered at 24 h post‐infection via IP injection. Blood was collected immediately before, and at 1, 3, 6, 12, 24 and 48 h post‐dose (three mice for pre‐dose, and three mice per dose per post‐dose time point). Plasma samples were prepared within 1 h of collection, stored frozen at −20°C before analysis. CD101 concentration in plasma was measured using liquid chromatography with tandem mass spectrometry (LC/MS/MS). Calibration standards were ranged from 2 to 10 000 ng/ml CD101 in plasma. PK parameters were calculated by non‐compartmental analysis using Phoenix WinNonlin (Pharsight, Mountain View, CA) software.

## Conflict of interest

DSP has received support from the US National Institute of Allergy and Infectious Diseases and has received support from Cidara, Astellas, Matinas and Merck, and participates in consult panels for these companies. YZ has received research support from Merck. GH, JBL, KB and VO are employees of Cidara Therapeutics, Inc.

## Supporting information


**Supplementary figure**. Echinocandin inhibition profiles of enriched GS complex from susceptible and resistant *C*. *albicans* and *C*. *glabrata* isolates. Each strain was tested in triplicate against both CD101 and MCF. Inhibition profiles were generated by using a normalized response (variable‐slope) curve fitting algorithm with GraphPad Prism, version 6.05, software (Prism Software, Irvine, CA)

Supporting info item
