# Supplementary material for: CD101: a novel long‐acting echinocandin
Source: Cell Microbiol. 2016 Jul 22;18(9):1308–16. doi: 10.1111/cmi.12640 (PMC5096055; doi:10.1111/cmi.12640)
Supplement: Supplementary file 1 — Supporting info item [file CMI-18-1308-s001.docx]

**Supplementary figure. Echinocandin inhibition profiles of enriched GS complex from susceptible and resistant *C. albicans* and *C. glabrata* isolates. Each strain were tested in triplicate against both CD101 and MCF. Inhibition profiles were generated by using a normalized response (variable-slope) curve fitting algorithm with GraphPad Prism, version 6.05, software (Prism Software, Irvine, CA).**
